# Multi-scale modelling reveals that early super-spreader events are a likely contributor to novel variant predominance

**DOI:** 10.1098/rsif.2021.0811

**Published:** 2022-04-06

**Authors:** Ashish Goyal, Daniel B. Reeves, Joshua T. Schiffer

**Affiliations:** ^1^ Vaccine and Infectious Diseases Division, Fred Hutchinson Cancer Research Center, Seattle, WA 98109, USA; ^2^ Department of Medicine, University of Washington, Seattle, WA 98195, USA; ^3^ Clinical Research Division, Fred Hutchinson Cancer Research Center, Seattle, WA 98109, USA

**Keywords:** COVID-19, SARS-CoV-2, novel variants, mathematical modelling

## Abstract

The emergence of new SARS-CoV-2 variants of concern (VOC) has hampered international efforts to contain the COVID-19 pandemic. VOCs have been characterized to varying degrees by higher transmissibility, worse infection outcomes and evasion of vaccine and infection-induced immunologic memory. VOCs are hypothesized to have originated from animal reservoirs, communities in regions with low surveillance and/or single individuals with poor immunologic control of the virus. Yet, the factors dictating which variants ultimately predominate remain incompletely characterized. Here we present a multi-scale model of SARS-CoV-2 dynamics that describes population spread through individuals whose viral loads and numbers of contacts (drawn from an over-dispersed distribution) are both time-varying. This framework allows us to explore how super-spreader events (SSE) (defined as greater than five secondary infections per day) contribute to variant emergence. We find stochasticity remains a powerful determinant of predominance. Variants that predominate are more likely to be associated with higher infectiousness, an SSE early after variant emergence and ongoing decline of the current dominant variant. Additionally, our simulations reveal that most new highly infectious variants that infect one or a few individuals do not achieve permanence in the population. Consequently, interventions that reduce super-spreading may delay or mitigate emergence of VOCs.

## 1. Introduction

The emergence of more infectious and lethal SARS-CoV-2 variants of concern (VOC) has dramatically extended the COVID-19 pandemic and contributed to multiple surges of infections and deaths across the globe. A better understanding of the epidemiological properties leading to the invasion and predominance of new VOCs may allow more strategic public health strategies to limit their future impact.

The *alpha* (B.1.1.7) SARS-CoV-2 variant was the first to demonstrate a significantly higher infectivity and virulence than baseline variants [1,2]. *Beta* (B.1.3.5.1) and *gamma* (P.1) variants also have slightly increased infectivity and virulence [3–5] as well as the ability to partially evade vaccine- or infection-induced immunologic memory [6–8]. The *delta* SARS-CoV-2 variant (B.167.2) capitalized on significantly higher transmissibility to quickly predominate in many countries including the United States [9]. The *omicron* variant recently outcompeted *delta* in South Africa and subsequently achieved global predominance. VOCs rapidly spread in unvaccinated groups but are also generally over-represented as ‘breakthrough’ infections of vaccinated individuals [10]. The detection of new more-transmissible variants continues to be delayed by sequencing limitations in many global infection hot spots.

Early during the pandemic, phylogenetic surveys identified population sweeps with variants containing single (or a few) point mutations [11]. Beginning in the summer of 2020, several variants emerged with an unexpectedly high number of new mutations (often greater than 12 in the genomic region encoding the viral spike protein [1,12,13]). Within-host evolution in immuno-compromised hosts is a plausible source for these variants as individuals with impaired immune function can shed virus at high viral loads for months, in the relative absence of selection pressure [14–17]. Some documented cases resulting in large numbers of mutations also involved possible incomplete selective pressure related to therapies.

Mathematical models are vital tools in infectious disease epidemiology [18]. Population genetic models with multiple strains and heterogeneous contact networks are extensively used to characterize epidemics [19–21]. Modelling has demonstrated that super-spreading events (SSEs) can play a particularly important role in emergence and spread for certain infectious diseases [22], particularly for coronaviruses with pandemic potential such as SARS-CoV-1, middle eastern respiratory syndrome epidemic (MERS) and SARS-CoV-2 [23,24]. Theoretical methods have been developed to characterize this phenomenon [25].

**Figure 1.**
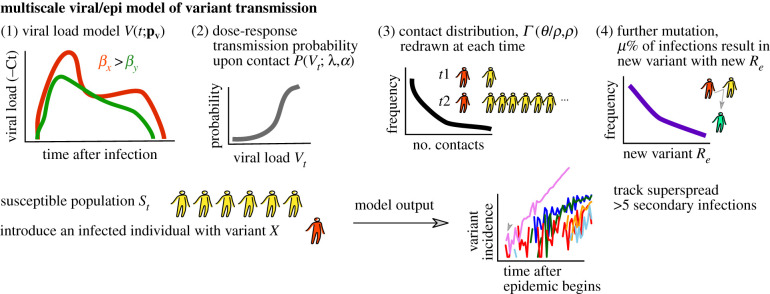
Multi-scale model schematic. We model an epidemic in which infected individuals with given variants (e.g. red) are introduced into a susceptible population (e.g. yellow). Individual viral load trajectories are tracked, and viral load is assumed to influence infection probability as a dose–response type function. Contacts occur stochastically over time and are drawn from a distribution in which potential SSEs (greater than five infections) are possible but not common. As incidence increases, additional variants each with potentially different transmissibility can emerge, though the depletion of susceptible individuals may influence these onward dynamics.

We developed a mathematical model that incorporates several crucial aspects of the SARS-CoV-2 pandemic: stochastic viral load-dependent transmission, non-homogeneous and time-varying contact networks for infected individuals, and emerging viral variants with heterogeneous infectivity (figure 1). Relative to prior models, we introduce a multi-scale approach that includes within-host viral dynamics. This addition serves two main purposes. First, it remains unclear whether variants with different viral load kinetics result in different epidemiological outcomes [26–29]. Second, once viral loads from an emerging variant are observed, viral properties (like infectivity on a per cell basis) can be quantified and used to project epidemiological outcomes. For example, it is now apparent that *in vitro* reductions in vaccine-induced antibody titer correlates with protection at an epidemiological scale [30].

While the COVID-19 pandemic has been consistently sustained by SSEs [31], our present work focuses on the connection between super-spreading and novel variant emergence and invasion. This analysis informs retrospective understanding of the global variant emergence patterns and the outsized benefit of targeting SSEs.

## 2. Results

### 2.1. Multi-scale mathematical model of SARS-CoV-2 variant spread in a population of individuals with time-varying and heterogeneous numbers of contacts

We previously constructed and validated a model that captures several quantitative features of SARS-CoV-2 transmission dynamics including that infected individuals have widely ranging viral loads throughout the time course of their infection, that viral loads tend to predict transmission, and that secondary transmission patterns are highly variable [31–33]. Super-spreading is common for SARS-CoV-2, and we defined super-spreading in our model by specifying events when a single-infected individual infects five other people within a day [34–36]. In some cases, we also applied a more stringent definition of SSEs as 10 or 20 secondary infections from a single-infected individual in a day.

We simulated scenarios by introducing an individual infected with a given variant into a susceptible population. This index case and any further cases had a viral load drawn from realistic distribution of within-host viral load parameters. Each case exposes a specific number of contacts during each time interval based on stochastic draws from an over-dispersed distribution. New variants emerge with a certain probability and the incidence of each variant is tracked. We followed variant incidence over time and assessed whether or which variants went *extinct*, and if any further new variants emerged. We also tracked the number of days between variant emergence in a single person and *invasion* (defined as 1000 cumulative infections). Any SSE was recorded. In simulations with co-circulating variants, we defined *predominance* when any new variant exceeded the baseline variant.

The main components of the multi-scale model are: (i) the viral load model, (ii) the transmission probability model, (iii) the exposed contact model, and (iv) the population/mutation model. A visual schematic of the model is in figure 1 and a complete mathematical exposition of the model is provided in the Methods.

First, the viral load model depends on the variant and is parameterized by several features of the within-host model (equation (4.1)) including viral infectivity and host response. Second, transmissibility is captured by mapping a viral load to a transmission probability in a dose–response manner (equation (4.2)). Thus, at any time *t* during the course of an individual's infection, they may have a different transmission probability that depends on their viral load at that moment. In addition to viral loads, the transmission probability is governed by two parameters in the dose–response model including the viral loads that corresponds to 50% transmission probability and the steepness of this dose–response curve. The modulation of these parameters is crucial to account for transmissibility differences beyond viral load. For example, some variants might in theory have enhanced aerosolization and/or the ability to bind the angiotensin converting enzyme 2 (ACE2) receptor of target cells more avidly such that lower viral loads might be compensated for by these factors, rendering the variant equally transmissible. The parameter values in the transmission model are provided in table 1.

**Table 1.** Parameter values for the multi-scale model. (The standard deviation of the random effects as estimated by a nonlinear mixed-effect model are provided in brackets () as described in [31].)

model	parameter	description	value and units
viral	log_10_*β*	viral infectivity	7.23 (0.2) virions^−1^ d^−1^
	*δ*	infected cell death rate	3.13 (0.02) d^−1^ cells^−k^
	*k*	innate immunity killing exponent	0.08 (0.02) unitless
	log_10_*π*	viral load corresponding to 50% infectiousness	2.59 (0.05) d^−1^
	*m*	viral load corresponding to 50% infectiousness	3.21 (0.33) d^−1^ cells^−1^
	log_10_*ω*	acquired immune recruitment rate	−4.55 (0.01) d^−1^ cells^−1^
	*r*	acquired immunity saturation exponent	10 unitless
	*δ_E_*	acquired immune removal rate	1 d^−1^
	*q*	precursor maturation rate	2.4 × 10^−5^ d^−1^
	*c*	viral clearance rate	15 d^−1^
	*T*(0), *I*(0), *M*_1_(0), *M*_2_(0), *E*(0)	host-cell initial conditions	10^7^, 1, 0, 0, 0 cells ml^−1^
	*V*(0)	virus initial conditions	$\frac{\pi I(0)}{c}$ copies ml^−1^
	ϑ	virus load scalar for additional variability simulations	1 unitless
transmission	*λ*	viral load corresponding to 50% contagiousness/infectiousness	10^7^ copies ml^−1^
	*α*	hill slope modulating contagiousness/infectiousness	10^7^ copies ml^−1^
	*τ*	average time delay for a newly infected person to begin producing virus	0.5 days
contacts	*θ*	average daily contact rate for *R_e_* = 1.0	2.3 d^−1^
		average daily contact rate for *R_e_* = 1.2	3.1 d^−1^
		average daily contact rate for *R_e_* = 1.4	3.5 d^−1^
		average daily contact rate for *R_e_* = 1.6	3.75 d^−1^
		average daily contact rate for *R_e_* = 1.8	4.0 d^−1^
		average daily contact rate for *R_e_* = 2.0	5.0 d^−1^
		average daily contact rate for *R_e_* = 2.2	5.5 d^−1^
	*ρ*	dispersion parameter	40

Third, we assumed that individuals have heterogeneous and time-varying numbers of exposed contacts. The underlying probability distribution used is a gamma distribution governed by the ‘super-spread parameter’ *ρ*. This value modulates the probability of a certain infected individual contacting with *n* others on any particular day (equation (4.3)). The choice of this distribution was justified by past epidemic data and by its inherent flexibility [22]. A low super-spread parameter means an infected individual is likely to transmit to the average number of secondary cases and resembles a normal distribution with low variability. High-contact dispersion on the other hand means that even though the mean transmission contacts is the same across times and individuals, most infected individuals contact 0 others, and a few contact many others (a fat tailed distribution). Previously, we estimated that the value was low (*ρ* = 0.1) for infections such as influenza in which there is low day-to-day and person-to-person variance in number of exposure contacts, and high (*ρ* = 40) for SARS-CoV-2 [31].

Fourth, we introduce infected cases into a large susceptible population. As more infections occur, susceptible individuals decrease, but prevalence is generally low enough that later variants are not significantly hampered by this depletion. We assume that a fixed proportion of newly infected individuals introduce further new variants. We used experimental data on ACE binding to estimate a distribution of within-host viral fitness that is roughly exponentially distributed [37], with an average below 1 such that most new mutations are neutral or deleterious and only a minor proportion are advantageous. However, because these are *in vitro* data focusing on simply one viral characteristic, not necessarily accounting for transmission probability, we also tested uniform distributions.

### 2.2. Global sensitivity analysis and the average effective reproduction number

The average effective reproduction number *R_e_* is a time-varying quantity calculated by summing over all secondary cases arising from single individual and averaging over all individuals during the simulation (see Methods). We assumed masking or other interventions that lower the transmission probability despite exposed contact with an infected individual [33,38] impact all co-circulating variants equally such that differences in *R_e_* between co-circulating variants are owing to their inherent parameters.

To simulate variants with different *R_e_*, we changed input parameters for viral variants (i.e. viral infectivity, viral production rate and immune modulation) and population behaviour (i.e. average number of exposure contacts and over-dispersion). To simplify our implementation, we sought to identify the most sensitive parameters influencing *R_e_* and modulate those to simulate new variants. Thus, we performed a global sensitivity analysis (see Methods) and found that the average number of contacts *θ* most strongly correlated with the average effective reproduction number regardless of changes in all other variables (partial rank correlation coefficient (PRCC) = 0.74, table 2). The second most influential parameter was the viral production rate *π*(PRCC = 0.64, table 2), emphasizing that the within-host model does affect between host dynamics. Notably, the dispersion parameter changed the variability but had little impact on the average reproduction number (PRCC = −0.02) in agreement with analytical calculations based on simpler models. Based on these results, when the average reproduction number was input into the model, it was through modification of the average number of exposure contacts.

**Table 2.** Relationship between model parameters and effective reproduction number inferred from global sensitivity analysis. (PRCC, partial rank correlation coefficient.)

parameter	description	range	PRCC with *R_e_*
*λ*	viral load corresponding to 50% infectiousness	[105, 108] copies ml^−1^	−0.27
*θ*	average number of contacts	[0.04, 40]	0.74
*ρ*	super-spread parameter	[0.4, 400]	−0.02
*β*	viral infectivity	[10^−9.2^, 10^−5.2^] virions^−1^ day^−1^	0.26
*δ*	infected cell death rate	[0.31, 5.0] day^−1^ cells^−k^	−0.19
*π*	viral burst size	[10^0.59^, 10^6.59^] virions cell^−1^	0.64
ϑ	viral load scalar	[0.1, 100]	0.26

### 2.3. Frequent stochastic extinction of new SARS-CoV-2 variants

**Figure 2.**
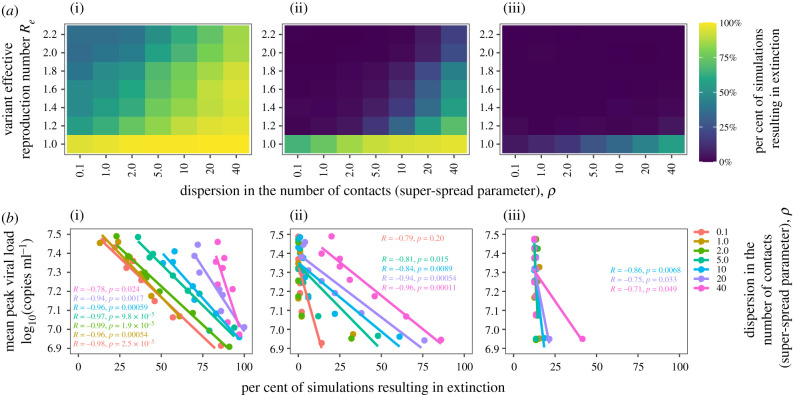
New SARS-CoV-2 variants with high transmissibility (*R_e_* > 1) often extinguish with low initial cases, but extinction is unlikely after an early super-spreading event. We simulated the introduction of 1 (i), 10 (ii) or 100 (iii) infected cases (columns) with a given variant into a population of 1 million susceptible individuals and allowed for time-varying viral load, stochastic transmission and super-spreading. (*a*) Heatmaps illustrate the percentage of simulations that resulted in extinction (blue: no extinction, yellow: frequent extinction) across ranges of super-spread parameter (gamma-distributed network dispersion) and variant reproduction number. Super-spread parameter ranges encompass low (*ρ* = 0.1, a realistic value for influenza) to high super-spread potential (*ρ* = 40, an upper estimate of SARS-CoV-2 infection). Ranges of effective reproductive number (*R_e_*) encompass values from throughout the COVID-19 pandemic, which can be modulated by factors such as circulating variant transmissibility, social distancing, masking and/or proportion immune at a given time. Note the electronic supplementary material, figure S1 shows that *R_e_* is not strongly influenced by the super-spreading parameter. (*b*) Correlation between extinction probability and peak viral load, coloured by super-spread parameter, illustrate viral load kinetics influence transmission dynamics, particularly for lower dispersion and single-case introduction. Here, peak viral load is a determinant of *R_e_*.

We identified that variant extinction is more likely when *R_e_* is lower but also when the contact network is highly over-dispersed as with SARS-CoV-2 (figure 2*a*). This suggests that most highly infectious SARS-CoV-2 variants will extinguish when generated within a single person, even when *R_e_* is quite high. We performed an equivalent analysis with 10 starting infections as might occur if an outbreak of a new variant first spreads in a small household or work cluster. The rate of extinction was still relatively high for low *R_e_* and high over-dispersion scenarios but decreased with higher *R_e_* values for a given variant. We next performed an analysis with 100 starting infections as might occur with a larger initial SSE or introduction of a new variant into a new region or country via travel. The rate of extinction was low for all *R_e_* values and assumptions regarding contact network dispersion. Therefore, although stochastic extinction of novel SARS-CoV-2 variants is likely to be common, once roughly 100 cases are established, a variant is likely to continue to expand exponentially in the absence of intensification of non-pharmaceutical interventions (NPIs) regardless of its ability to generate SSEs.

As a check, simulation of our multi-scale model with 1 initial case and *ρ* = 1 were in relatively good quantitative agreement with analytical results from epidemiology and population genetics studies showing that the probability of non-extinction is the inverse of the reproductive number or the selection coefficient [18,39]. For example, *R_e_* of 2 leads to approximately a 50% chance of burnout (figure 2*a*).

We next quantified the impact of peak viral load on variant extinction (figure 2*b*). Assuming a single initial infection, even variants with higher peak viral load had only a modest (approx. 20%) chance of survival when the contact network was highly over-dispersed, which contrasts sharply with variants that had lower dispersion of susceptible contacts. Starting with 10 or 100 initial cases again allowed for a higher chance of survival of new variants; however, the effect of peak viral load on the probability of extinction was much greater as the number of starting infections increased in the case of a highly over-dispersed virus (pink line, *ρ* = 40). Thus, slight decreases in viral transmissibility, here represented by a decrease in average peak viral load, would increase extinction probability of new SARS-CoV-2 variants [40]. This suggests that vaccines or treatments in the form of post-exposure prophylaxis that blunt peak viral load even slightly could not only lower the chances of the emergence of new viral strains in the population, but also break the chain of transmission after the variant is established in more than 10 people.

### 2.4. Highly variable timing of SARS-CoV-2 variant invasion at realistic effective reproductive numbers

**Figure 3.**
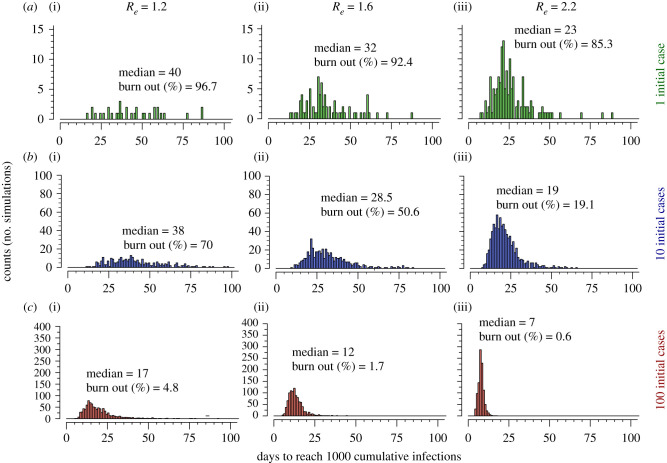
Time to invasion among SARS-CoV-2 simulations which do not burn out. Invasion is defined as 1000 cumulative infections. Scenarios (*a*–*c*) modulate the number of initial cases (1, 10 and 100) and columns modulate the effective reproductive number of the initial variant, respectively. Low *R_e_* and low number of initial cases (*a*(i)) is associated with a higher median and larger variance in time to invasion. Differences in *y*-axis scale through rows highlight that there is less extinction in scenarios with higher numbers of initial cases and higher *R_e_*.

We next evaluated time from first case of a new variant to invasion (defined as 1000 cumulative infections) in simulations in which stochastic burnout did not occur. We performed 1000 simulations under each assumed value of *R_e_*. When starting with one infection, we observed a wide variance in time to invasion with an increase in the median time from 23 to 40 days as *R_e_* decreased from 2.2 to 1.2 (figure 3). The variance and median time (19 to 38 days) to 1000 infections were similar when starting from 10 infections (figure 3*b*) but the median time (7 to 17 days) and variance decreased when starting from 100 infections (figure 3*c*) demonstrating that stochastic forces are less important once 100 cumulative infections are reached. To check that simulation size was not a factor in results, we found similar results using two examples of low and high *R_e_*, and 1 initial case with 10 000 simulations (electronic supplementary material, figure S2A).

### 2.5. Contribution of early super-spreader events to rapid variant invasion

**Figure 4.**
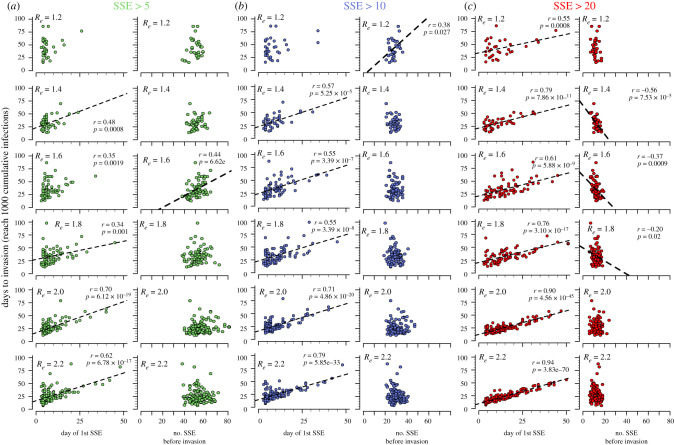
Relationship between timing and number of SSEs and time to invasion. Invasion is defined as 1000 cumulative infections. Scenarios assume varying definitions of SSEs as (*a*) greater than 5, (*b*) greater than 10 and (*c*) greater than 20 secondary infections on any day. Each plot also varies the invading variant reproductive number (noted in bold *R_e_*). Correlations were tested between the day of the first SSE as well as the number of SSEs against the time of invasion (defined as reaching 1000 cumulative infections). An early day of the first SSE predicts more rapid time to invasion, particularly when the invading variant had higher *R_e_* and an SSE is defined as greater than 20 infections in a day (see lower red panels). The total number of SSEs was generally not predictive of time to invasion. Pearson correlation coefficient (*r*) and corresponding trendlines are only noted in plots for which *p* < 0.05.

We next examined the timing and number of SSEs during these simulations. SSEs were variably defined as events in which one individual infected at least 5 (figure 4*a*), 10 (figure 4*b*) or 20 (figure 4*c*) others in a day. With each definition and across all assumed values of *R_e_* = 1.6 or higher, the timing of the first SSE correlated with time to invasion. The strength of this correlation generally increased as the definition of a SSE became more stringent and with higher values for *R_e_* (figure 4).

The number of SSEs prior to invasion was generally not positively correlated with time to invasion at high *R_e_* values, signifying that multiple small SSEs do not strongly accelerate invasion. Exceptions were at lower values of *R_e_* with events defined as greater than 5 or greater than 10 infections. Number of SSEs correlated negatively with time to invasion assuming low to moderate values for *R_e_* (1.2–1.8) and less inclusive definitions of SSEs (at least 20 secondary infections, figure 4*c*). To check that this simulation size was sufficient, we demonstrated similar results with 10 000 simulations in the most stochastic regime: low and high *R_e_*, and 1 initial case (electronic supplementary material, figure 2*B*).

SSEs were associated strongly with variant invasion. Simulating variants with *R_e_* = 1.2 with a single initial case, 95 out of 1000 simulations had an SSE with greater than five infections, out of which 33 reached the invasion threshold of 1000 cumulative infections. Across simulations without an SSE, no invasion was observed (0 out of 905, *p* < 1 × 10^−16^, Fisher's exact test). The low probability of invasion without an SSE was observed for all assumed values of variant *R_e_*. While our prior results, demonstrate that high *R_e_* is a key determinant of variant invasion (figure 1a), these simulations with parameter assumptions compatible with known features of variants B.1.1.7 and B.167.2 [1] show that early SSEs are also key drivers of variant invasion and predominance.

### 2.6. Increased likelihood of variant invasion when circulating baseline variants have an effective reproductive number less than or equal to one

**Figure 5.**
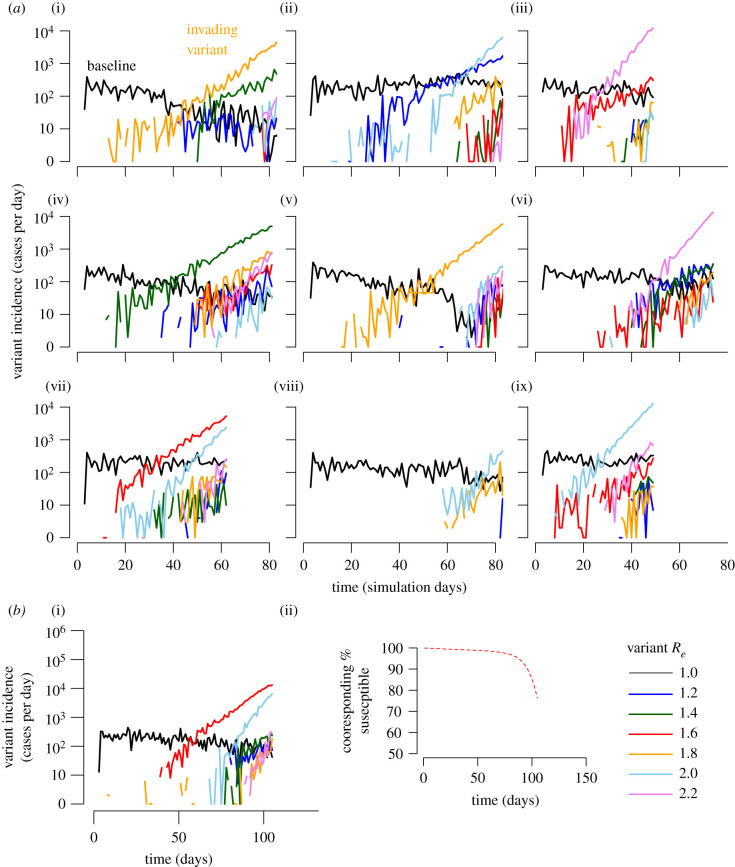
New variant predominance depends on timing of variant introduction, variant effective reproductive numbers and numbers of ongoing infections. (*a*) Nine out of 100 simulations starting with 1000 infections of a baseline variant (black line) with *R_e_* = 1 are shown. New variants (e.g. orange line in (i)) are randomly generated in 1% of transmissions and coloured according to *R_e_* (drawn from a uniform distribution in bins of 0.2). The first new variant to reach 10 cases per day usually predominates though occasionally subsequent more-transmissible variants will expand and reach predominance (light blue in (ii)). Third-generation new variants become much more common as second-generation variants increase the total number of circulating infections (e.g. many lines in (iv, vi) after day 50). (*b*) A single example of a simulation with 1000 infected individuals but tracking until 250 000 cumulative infections admits similar kinetics and demonstrates only minor depletion of susceptible individuals.

We next performed simulations testing the probability of novel variant invasion given a circulating variant already infecting 1000 individuals. We first assumed that novel variants emerge probabilistically, with 1% of transmission events resulting in novel variant introductions. We restricted our analysis to consider scenarios with fit variants, initially allowing effective *R_e_* for the new variant to be uniform between 1 and 2.2 at intervals of 0.2. Figure 5*a* shows nine examples of simulation trajectories. For each value of baseline *R_e_*, we performed 100 simulations until 100 000 cumulative infections were generated or until stochastic burnout of all variants occurred. Additionally, figure 5*b* shows similar patterns for a simulation performed starting with 1000 infected individuals but tracking until 250 000 cumulative infections and illustrates that these trajectories occur with marginal depletion of susceptible individuals, meaning competition is probably not a large factor in dynamics of new variant emergence and invasion. These trajectories showed that more infectious variants (higher *R_e_*) were more likely to invade, though the timing of invasion can be highly variable owing to stochasticity and super-spreading.

### 2.7. High-incidence outbreaks and formation of third-generation variants

In simulations with the baseline variant *R_e_* = 1.0, new high-incidence waves of infections with second-generation variants were predictably associated with the emergence of novel third-generation variants—some of which ultimately predominated owing to higher *R_e_* (figure 5*a*, e.g. top middle light blue line). This finding highlights that new variant emergence and invasion might be limited by maintaining lower incidence, though this conclusion requires further empirical validation.

### 2.8. More infectious invading variants relative to circulating variants

**Figure 6.**
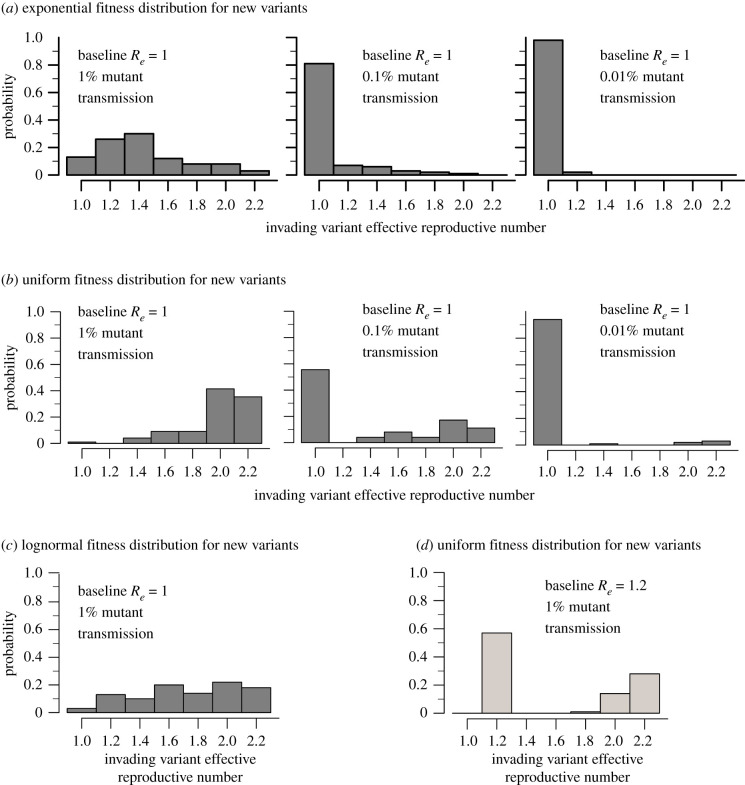
Histograms of *R_e_* of invading variants. Given the assumption that 1%, 0.1% and 0.01% of new infections result in transmission of a new variant, we calculated the probability of predominance of new variants with different fitness drawn from an exponential (*a*), uniform (*b*) or lognormal (*c*) distribution. A lower frequency less than 1% of new variant introductions increased the likelihood that the baseline variant with *R_e_* = 1 continued to predominate. (*d*) If the baseline variant had *R_e_* = 1.2, it had a much higher chance (approx. 50%) of remaining dominant even with 1% variant generation unless the emerging variants had high transmissibility (i.e. *R_e_* > 1.8).

We explored the *R_e_* of invading variants based on different assumptions of the distribution of new variants created by mutation during the simulation. Assuming 1% of transmission events result in a new variant, drawing these variants from an exponential (mean = 1, figure 6*a*), uniform (figure 6*b*) or lognormal (mean = 1, figure 6*c*) fitness distribution lead to slightly different outcomes for *R_e_* of invading variants with the uniform distribution favouring more fit viruses. When we assumed a baseline variant with *R_e_* = 1.0 and a new variant created in 1% of transmission events with a uniform distribution, we observed predominance of a second and third-generation variant in 98 out of 100 simulations, whereas if mutation occurred in 0.1 or 0.01% of transmission events, then variant takeover only occurred in 44% and 6% of simulations. When variants with a new *R_e_* were generated more often, there were more evenly distributed values of emerging *R_e_*.

We next performed 100 simulations using a more fit baseline variant with *R_e_* = 1.2 and 1% of transmission events resulting in a new variant (figure 6*d*). Variant invasion only occurred in 42 out of 100 simulations and invading variants had high *R_e_* > 1.8. Simulations with a baseline variant with *R_e_* = 0.8 allowed variant invasion in all 100 simulations (conditioned on no epidemic burnout, data not shown). This result helps explain why new variant predominance is often observed when incidence of the baseline variant is decreasing [1].

## 3. Discussion

Our modelling illuminates an underappreciated determinant of novel SARS-CoV-2 variant emergence and invasion. Intuitively, variant invasion becomes more likely if that variant has inherently higher transmissibility, a result supported by our modelling. Yet, because of the stochasticity inherent in over-dispersed contact networks, high transmissibility does not guarantee invasion. Our results suggest that most new highly infectious variants which emerge from infected individuals never spread substantially in the population. It also raises the provocative hypothesis that other human coronaviruses with pandemic potential (e.g. SARS, MERS and SARS-CoV-2) are introduced into the human population commonly. Pre-emptive public health efforts are justified to mitigate as many of these events as possible.

Previous works using epidemiological models have studied the balance between transmissibility and over-dispersion [22,24]. Our results agree with findings from classic epidemiology, which illustrate stochastic extinction can occur in an SIR/SIS system even given a transmissible pathogen with an effective reproductive number (*R_e_* > 1) [18,41,42]. The over-dispersed secondary infection rate associated with SARS-CoV-2 only increases the likelihood of stochastic extinction [22,43]. Some analytical works addressing the complexities of extinction on networks with over-dispersion have been performed [44]. For realistic approximation of SARS-CoV-2 transmission, viral load kinetics influence transmission probability [31,33,45,46]. Therefore, to integrate relevant biologic and epidemiological processes, we developed a multi-scale model that agrees with the main conclusions of past works, while allowing for direct connections between measurable viral dynamic properties, super-spreading and multi-variant epidemiological dynamics such as novel variant invasion.

Among variants that emerge in a single person, our model suggests that, in addition to viral transmissibility, early SSEs, particularly those involving more than 20 people may dictate whether invasion or extinction occurs. Super-spreading events provide a head start for a variant, bypassing the slower early phase of exponential growth into rapid deterministic growth [47]. SSEs later during an epidemic growth curve are relatively less important for a variant to achieve predominance.

From a public health perspective, our results provide yet another reason to intensely focus NPIs on preventing large SSEs. This policy prescription includes the prohibition of large indoor gatherings among unvaccinated people, a focus on adequate ventilation in indoor work environments and schools, and enforcement of highest quality masks (K95 or N95) in circumstances where high-risk group exposures cannot be avoided [33]. Prevention of SSEs will limit number of infections, lower the introduction of new variants and decrease the probability that a single large SSE will initiate a more rapid local epidemic as has already been documented in Boston, South Korea and multiple other locations during the pandemic [48,49]. As incidence increases during a local outbreak, the probability of a super-spreading event continually increases, making these interventions more difficult, which re-emphasizes public health efforts to keep cases low.

Our model has important limitations. While the model's qualitative findings are robust, we cannot estimate the outbreak size and viral transmissibility that combine to guarantee new variant emergence or invasion. These quantities depend exquisitely on specific local epidemic parameters that are not typically known. For instance, it is not yet clear whether the percentage of immuno-compromised hosts varies across populations, which may be based on factors such as HIV prevalence and availability and/or use of immunosuppression for organ transplantation and cancer treatment. The number of secondary infections created by a person with new variants may also differ from that of other members of the population in ways that are difficult to project. On the one hand, these individuals may shed for longer and at a higher viral load [14,15]. Yet, they also may be more ill and therefore quarantined at home or in the hospital limiting contact exposures. Moreover, while all variants are probably impacted in the same way by the introduction of NPIs such as masking and physical distancing, the use of these interventions varies considerably among regions and over time. Animal reservoirs for SARS-CoV-2 have been recently detected [50], and the dynamics of transmission between animals and humans remains unknown.

Novel variant reproductive numbers (*R_e_*) in this model are also associated with invasion probability. Importantly here, *R_e_* represents only enhanced transmissibility given a certain level of population immunity rather than more complex phenomena such as a change in asymptomatic fraction. Analyses incorporating such phenomena would require merging our model with a detailed epidemiological model, beyond the scope needed to illustrate the strong impact of super-spreading. Moreover, increased transmissibility for novel variants was demonstrated by modelling and *in vitro* experiments showing enhanced binding to the ACE2 receptor in respiratory cell lines [1,5].

In summary, new variants are likely to be frequently created and introduced into the population during large waves of SARS-CoV-2 infection. Yet even transmissible variants often undergo stochastic extinction and those that ultimately invade are often associated with early SSEs. When the dominant variant is decreasing, this represents a delicate period in which new variants are more likely to take hold. However, decreasing incidence reduces the probability of SSEs. Overall, our work adds to powerful existing rationale to make all efforts to reduce SSEs through mass vaccination and strategic continued use of NPIs [23].

## 4. Methods

### 4.1. Data-validated SARS-CoV-2 within-host model captures viral loads over time

We used the within-host model describing the SARS-CoV-2 infection from our previous study [32]. This model assumes that the contact of SARS-CoV-2 (*V*) with susceptible cells (*T*) produces infected cells at rate *βVT* which then generates new virus at a per capita rate *π*. The model also incorporates the death of infected cells mediated by the innate responses (modelled as a density-dependent killing term: *δI^k^*) and the explicitly modelled acquired immune response of SARS-CoV-2-specific effector cells *E*(*t*). The Hill coefficient *r* allows for nonlinearity and saturation in acquired immune killing. The parameter *ϕ* defines the saturation level: the concentration of SARS-CoV-2-specific effector cells at which the killing of infected cells becomes half-maximal. In the model, the rise of SARS-CoV-2-specific effector cells is described in two stages. The first stage defines the proliferation of a precursor cell compartment (*M*_1_) at rate *ωIM*_1_ and differentiation into a secondary precursor cell compartment (*M*_2_) at a per capita rate *q*. Finally, second precursor cells differentiate into effector cells at the same per capita rate *q* and are cleared at rate *δ_E_*. Other models have described within-host viral loads differently, including by using multiple compartments and without an adaptive immune response [51–53]. Against our data, this model was optimally parsimonious, so we continue with it here. The model is expressed as a system of ordinary differential equations with time derivative denoted by the overdot:

4.1 $$\begin{array}{rl} \dot{T} & =-\beta VT,\\ \dot{I} & =\beta VT-\delta I^{k}I-m\frac{E^{r}}{E^{r}+\phi^{r}\;}I,\\ \dot{V} & =\pi I-\gamma V,\\ {\dot{M}}_{1} & =\omega IM_{1}-qM_{1},\\ {\dot{M}}_{2} & =q(M_{1}-M_{2})\\ \mathrm{and}\;\;\;\dot{E} & =qM_{2}-\delta_{E}E. \end{array}\}$$

### 4.2. Dose–response model linking viral load to secondary infection probability

We also employed our previously developed ‘dose–response’ model to estimate both contagiousness *P*_contagion_ (the probability of effective exposure) and infectiousness *P*_infect_ (the probability of cellular infection) as functions of transmitter viral load [31]. Each of these mechanistic processes was modelled with an identical Hill function such we link the viral load of a transmitter *V*(*t*) (dose) to the probability of effective exposure and/or cellular infection (response) as

4.2 $$P_{\mathrm{contagion}}=P_{\mathrm{infect}}=\frac{V{\left(t\right)}^{\alpha }}{\lambda^{\alpha }+V{\left(t\right)}^{\alpha }},$$

where *λ* is the viral load that corresponds to 50% infectiousness and/or 50% contagiousness and *α* is the Hill coefficient that controls the sharpness in each dose–response curve.

### 4.3. Transmission model and effective reproduction number

As in our previous model [31], we determined the total exposed contacts of a transmitter within a time step (Δ*t*) using a gamma distribution, i.e.:

4.3 $$\eta_{\Delta t}∼\Gamma \left(\frac{\theta }{\rho },\;\rho \right)\Delta t,$$

where *θ* and *ρ* represent the average daily contact rate and the dispersion parameter, respectively. The true number of exposure contacts (with viral airway exposure) was then obtained by multiplying the total exposed contacts and the contagiousness of the transmitter. We modelled infectiousness as a Bernoulli event with mean *P*_infect_, yielding the number of secondary infections within a time step as

4.4 $$Y_{\Delta t}=\mathrm{Ber}(P_{\mathrm{infect}})P_{\mathrm{contagion}}\eta_{\Delta t}.$$

Finally, we summed up the number of secondary infections over 30 days since the time of exposure to obtain the individual effective reproduction number, which we denote with a lower case variable for each individual:

4.5 $$r_{e}(i)=\underset{\Delta t}{\sum }Y_{\Delta t}.$$

The total effective reproduction number is then the average of the individual reproduction number taken over all infected individuals currently in the simulation:

4.6 $$R_{e}=\frac{1}{n}\underset{i}{\sum }r_{e}(i)\;.$$

In simple steps, we followed the procedure below to estimate *R_e_*:

1. simulate viral load *V*(*t*) of a simulated infected individual using the within-host model;
2. for a given combination of (*λ*, *τ*, *α*, *θ*, *ρ*):
   (a) for each time step Δ*t*:
    (i) compute *P_t_*[*V*(*t*);*λ*, α];
    (ii) draw $\eta_{\Delta t}∼\Gamma ((\theta S_{t})/(\rho S_{0}),\rho )\Delta t$;
    (iii) calculate $Y_{\Delta t}=\mathrm{Ber}(P_{t})P_{t}\eta_{\Delta t}$, where *P_t_* = *P*_infect_;
   (b) calculate $R_{e}=\sum_{\Delta t}Y_{\Delta t}$;
3. repeat steps 1 and 2 to estimate *R_e_* for 1000 infected individuals. The population level *R_e_* can then be calculated by taking the mean of 1000 individual *R_e_* values.

### 4.4. Population simulation

Armed with the model for an individual, we next simulated temporal transmission throughout a population. For each successful transmission, we assumed a slight delay of *τ* days for the first infected cell to produce virus. A key change was made to the previously published model to improve its realism: we allow for the depletion of susceptibles as the simulation proceeds such that later variants do have a slight disadvantage even if they are intrinsically more fit. In the procedure above, *S_t_* is calculated at each time *t* beginning with an initial population of 1 million susceptible individuals. We followed the procedure below to transform our previously published transmission model into multi-class temporal transmission model:

1. discretize the time-space of 150 days over time steps Δ*t* of 1 day;
2. with *n_tc_* representing the number of transmitters at any time *t* of variant ‘*c*’, we start with presumed *n_oc_* transmitters at *t* = 0 of variant *c* and zero transmitters at the remaining time points for all variants;
3. starting at *t* = 0, for each of the seven variants:
  (a) we determine the number of transmitters (infected individuals) at that time step of variant ‘*c*’ and then,
  (b) for *i*th of *n_tc_* transmitters:
    (i) simulate *V_i_*(*T*) over [*t*, *t* + 30] at daily intervals (i.e. Δ*T* = 1) using the within-host model in equation (4.1);
    (ii) compute *P*_infect,*i*_[*V_i_*(*T*);*λ*, *α*];
    (iii) draw $\eta_{\Delta T,i}∼\Gamma ((\theta S_{t})/(\rho S_{0}),\;\rho )\Delta T$;
    (iv) calculate *Y*_Δ_*_T_*_,*i*_ = *Ber*(*P_t_*_,*i*_)*P_t_*_,*i*_*η*_Δ_*_T_*_,*i*_, where *P_t_*_,*i*_ = *P*_infect,*i*_;
    (v) determine times of successful transmission (*t_s_*) as those times ‘*t*’ where *Y*_Δ_*_T_*_,*i*_ > 0 and the number of secondary transmissions at those time points as *Y*_Δ_*_T_*_,*i*_;
    (vi) determine which strain was transmitted at times of successful transmission using *μ_T_*_,*i*_ = Ber(*μ*). If *μ_T_*_,*i*_ equals 1, then only a mutant strain is transmitted and the class of the mutant strain is randomly selected from seven pre-specified variants;
    (vii) update *n_tc_* = *n_tc_* + *Y*_Δ_*_T_*;
4. repeat step 3 for *t* = Δ*t*, *t* = 2Δ*t* and so on over the discretized time-space of 150 days.

Since we follow the transmission dynamics from each infected individual and the number of secondary transmission per day (i.e. *Y*_Δ_*_T_*_,*i*_), the SSE can be simply characterized by *Y*_Δ_*_T_*_,*i*_ > SSE_threshold_, where SSE_threshold_ is the SSE threshold. For example, SSE_threshold_ takes the value of 5, 10 and 20 secondary transmissions in figure 4.

### 4.5. Global sensitivity analysis

We tested the sensitivity of the calculation of *R_e_* on seven parameters from all parts of the multi-scale model: λ, θ, ρ, β, δ, π and ϑ . These parameters represent 50% infectiousness, the average number of exposure contacts, over-dispersion parameter, viral infectivity, the death of infected cells mediated by innate responses, viral production rate and increase in viral loads for other unaccounted reasons (i.e., V(T)=ϑ V(T), respectively. We varied *λ*, *θ*, *ρ*, *β*, *δ*, *π* and ϑ in plausible ranges (table 2), and using Latin hypercube sampling, we next generated 1000 parameter combinations and calculated *R_e_* for each parameter combination using the procedure described in *Population simulation*. The PRCCs were calculated for all seven parameters, table 2.

### 4.6. Simulating multi-class temporal dynamics from the transmission model

To simulate multi-variant dynamics, we assumed seven classes of mutant strains, each with a different *R_e_* of 1.0, 1.2, 1.4, 1.6, 1.8, 2.0 and 2.2 which were modelled by adjusting *θ* of 2.3 d^−1^, 3.1 d^−1^, 3.5 d^−1^, 3.75 d^−1^, 4.0 d^−1^, 5.0 d^−1^ and 5.5 d^−1^, respectively. In the case of *R_e_* = 0.8 (simulated with *θ* = 1.1 d^−1^), we allowed for eight variants instead of seven.

### 4.7. Parameter values

For all simulations, we used parameter values from table 1. Viral parameters taken derived from a nonlinear mixed-effect model fitted to human viral load data as described in [31]. Some parameters are changed within their standard deviation to allow variability in the viral dynamics including the peak viral load and the duration for which an individual maintains infectious levels of viral loads. Transmission and contact parameters were estimated in that work by comparison to empirically observed individual *R_e_* and serial interval histograms as well as mean *R_e_* across individuals (R_0_ ∈ [1.4 2.5]) and mean serial interval across individuals (SI ∈ [4.0 4.5]) early during the pandemic [34,35,54–56].

## Data Availability

All code and data to reproduce results in the work can be accessed at https://github.com/ashish2goyal/Pandemic_temporal_simulation_with_superspreader_events.
